# Plant–pollinator interactions and floral and nectar traits shape the diversity of the nectar mycobiome

**DOI:** 10.1038/s41598-026-42903-3

**Published:** 2026-03-12

**Authors:** Kamil Kisło, Marcin Mazurkiewicz, Bartłomiej Starzyński, Marcin Zych, Mikołaj Wołącewicz, Andrzej Bajguz, Magdalena Chmur, Katarzyna Roguz

**Affiliations:** 1 Faculty of Biology, University of Warsaw, Warsaw, Poland; 2 Faculty of Biology, University of Białystok, Białystok, Poland; 3 Marburg University, Department of Biology, Evolutionary Ecology of Plants, Marburg, Germany

**Keywords:** Amino acids, Nectar sugar, Fungi, Nectar fungal communities, Fructose, Glucose, Microorganism, Ecology, Ecology, Microbiology, Plant sciences

## Abstract

Beyond its essential role in plant–pollinator interactions, floral nectar serves as habitats for diverse fungal communities that can influence plant–animal mutualism. Although it has long been known that fungi can reach high densities in floral nectar, the factors structuring their diversity remain poorly understood. In this study, we evaluated how the presence of mycobiome influences nectar properties and how floral traits, nectar chemistry, and flower visitors’ presence shape nectar-inhabiting fungi diversity. We examined ten insect-pollinated plant species from nine families, encompassing a broad range of floral morphologies and flower visitor interactions. We recorded differences in sugar and amino acids presence in flowers with open and restricted visitors access. These differences are likely linked to the presence and activity of mycobiome introduced primarily through animal visits. Our results indicate that fungal diversity in nectar is associated with the presence of specific sugars, particularly fructose and glucose. In contrast, we found no significant relationships between fungal diversity and amino acid composition, floral traits, or flower visitor group. The generally weak effect of flower visitors on fungal diversity may be partially explained by a dilution effect in highly diverse floral environments, where microbial inoculation is distributed across many plant hosts.

**Supplementary Information:**

The online version contains supplementary material available at 10.1038/s41598-026-42903-3.

## Introduction

Flower nectar provides a diverse and often very demanding habitat for mycobiome^1–5^. Although it has long been known that fungi can reach high densities in nectar^2,6–9^, relatively little is understood about the factors that influence and structure their diversity. Available studies indicate that the composition of nectar mycobiome is shaped by a dynamic interplay of opposing forces. On one hand, nectar offers a nutrient-rich environment that supports growth due to its high energy content and relatively easy access. On the other hand, its high osmotic pressure, frequent presence of antifungal proteins, and the short lifespan of individual flowers act as strong ecological filters, limiting the number of fungal species capable of establishing and persisting in this environment^1,3,4,7–10^. Nectar constitutes a highly dynamic microhabitat — it is a subject to evaporation under high temperatures, dilution by rain, and depletion through repeated pollinator visits or nectar robbers, which further contributes to its challenging and selective nature for microbial colonizers [e.g., 1,6–9]. Consequently, only a limited number of fungal species’ diaspores can reach nectar and not all that arrive there are able to colonize it successfully^7^.

Nectar-dwelling fungi, primarily yeasts, are frequently engaged in competitive interactions for limited resources within nectar thus the composition is typically species-poor and dominated by a few cosmopolitan genera^4,11–13^. Among the most common and widely distributed species are *Metschnikowia rancensis*, *M. gruessii*, and *Starmerella bombicola*, which are recognized for their ability to dominate nectar fungal communities and adapt to diverse host plants^8,14,15^. *Zygosaccharomyces rouxii*, another notable taxon, is known for its extreme osmotolerance, and is frequently encountered in nectar and bee-associated habitats^15^. As nectar is also often colonized by bacteria, which can reach high densities, fungi interact with them through competition for resources, production of inhibitory compounds, or modification of nectar properties such as pH, sugar composition or secondary metabolites^3,16,17^. These microbial interactions may further influence which fungal taxa are able to establish and how their communities are structured. Altering other nectar properties, like sugar and amino acid content and composition^8,18,19^, may shape cues important for animal attraction in service-resource mutualisms^12^.

Certain floral traits can directly influence the presence of fungi in nectar. For example, whether nectar is exposed or protected by floral structures, and whether flowers are pendant or horizontally oriented, may affect the likelihood of airborne microbial colonization. A study on two Spanish plant species, *Digitalis obscura* and *Atropa baetica*, found that basidiomycetous yeasts, such as *Cryptococcus* spp., were likely introduced into nectar via airborne dispersal^2^. Nectar presentation can also mediate the impact of environmental stressors such as UV radiation, desiccation, and nutrient patchiness, all of which may limit mycobiome establishment^20^.

Floral traits can also indirectly influence the nectar mycobiome, for example by shaping flower visitors’ community and behavior. Traits involved in pollinator communication, such as flower size, shape, and arrangement on the stem, play a particularly important role in determining which animals visit flowers and how frequently^20–22^. These interactions, in turn, affect the composition and dispersal of fungi in nectar, as it is often a subset of organisms found on visitors’ bodies^5,23^. For example, larger flowers or inflorescences are often more visually prominent and may signal greater reward availability^24^, attracting both pollinators and herbivores^25–27^. There are also studies showing that flower size may influence the likelihood of pollinators defecating on floral surfaces, which can in turn affect mycobiome diversity^28^.

Beyond flower traits, also flower nectar plays a crucial role in shaping the nectar mycobiome. Although ecological studies have mostly focused on nectar role from the perspective of animal consumers e.g^29–31^; it is also widely exploited by specialized microbial communities e.g^1,32,33^.. While nectar composition varies widely among species, it is essentially a mixture of sugars and water^34^ often supplemented with other compounds such as amino acids^35^. It is assumed to be initially sterile; while newly opened flowers are not^3,4^. Therefore, nectar is often rapidly colonized by flowers’^5^ and visitors’ microbiome^8,36–38^.

While floral nectar is a food for pollinators, for nectar-inhabiting fungi it represents a vital and highly variable habitat that strongly influences and filters colonization, survivability, growth and among taxa interactions^3,4,32,35,39,40^. High osmolarity^7^ and unfavorable biochemical conditions^41^ can limit fungal colonization and survival. In particular, high sugar concentrations have been shown to reduce diversity by limiting the success of fungi unable to tolerate or metabolize such environments^2,42^. Interestingly, many microorganisms appear to be unaffected by nectar compounds traditionally considered inhibitory. For example, the nectar-specialist yeast *M. reukaufii* shows no significant response to most nectar secondary compounds^11^. Also the presence of nectar-inhabiting fungi can substantially alter nectar properties by metabolizing sugars, often reducing total sugar concentrations and changing the glucose-to-fructose ratio^5,8,43^. Some yeasts and fungi can also modify amino acid profiles by either consuming available amino acids or releasing metabolic byproducts, thereby affecting the nutritional balance of the nectar^44^.

By producing and preserving nectar, and by attracting and provisioning pollinators, floral traits and nectar properties play a central role in shaping the composition and dynamics of the nectar mycobiome. Despite the vital role of plant-pollinators-microbiome interaction in the ecosystems, surprisingly little is known about the role of flower traits and reward properties in shaping nectar microbiome diversity. The presence of microorganisms in flower nectar is common, and its role extends from modifying the reward properties, through the alteration of pollinators’ behavior to improving plant and pollinators’ health^5,45,46^. The interconnectedness between human, animal, and environmental health calls for microbiome stewardship — a careful and responsible management of ecosystem resources using the microbiome^47^. Global biodiversity should shift our attention to the potential of microorganisms in improving ecosystem resilience and restoring their crucial functions^5,44,45,48,49^. Pollination is one of such important ecological functions affected by nectar microbial communities that interfere with pollination efficiency, plant and pollinator fitness. The more we know about the factors influencing plant-pollinator-microbiome interaction, the better equipped we are to protect it.

To evaluate the effect of floral traits, nectar properties and plant-pollinator interactions we used ten insect-pollinated plant species from nine families. In selected plant species we recorded flower traits important for microbial maintenance and interaction with flower visitors, as well as we analyzed nectar properties and recorded flower visitors. We aimed to assess (1) how floral traits and nectar properties shape nectar mycobiome diversity, (2) the relationship between the mycobiome and nectar properties and (3) the role of flower visitors in shaping nectar mycobiome diversity.

## Materials and methods

The study comprised two stages: fieldwork and laboratory analysis. Field data were collected from June to August 2022 at the University of Warsaw Botanic Garden (Poland), while laboratory work was conducted at the Faculty of Biology, University of Warsaw (Poland), in autumn 2022. Ten plant species from the Botanic Garden collection were selected based on nectar availability and floral traits potentially influencing mycobiome diversity (e.g., size and orientation). Flowers were categorized into three size classes: small, medium, and large, based on flower diameter. Based on the orientation on the stem, flowers were divided in three groups representing species with pendant, upward facing and arranged at the right angle on the stem. The following plant species representing native and non-native ornamental species were finally selected: *Angelica archangelica* (Apiaceae), *Iris pseudacorus* (Iridaceae), *Polemonium caeruleum* (Polemoniaceae), *Impatiens walleriana* (Balsaminaceae), *Kniphofia uvaria* (Asphodelaceae), *Phlomis russeliana* (Lamiaceae), *Lysimachia clethroides* (Primulaceae), *Salvia hybrida* (Lamiaceae), *Lilium martagon* (Liliaceae) and *Dianthus seguieri* (Caryophyllaceae). The list of plants with their characteristics is available in Table 1 (Supplementary materials).

### Nectar collection

Before nectar collection, half of the flowers or inflorescences were covered at the bud stage with nylon mesh to prevent pollinator access. When the flowers reached anthesis, between 7:00 and 11:00, all available nectar was collected using sterile pipette tips and sterile microcapillaries. For each species, we collected at least 30 nectar samples — 15 from isolated and 15 from open flowers. When flower availability permitted, the entire nectar sample was collected from a single flower (*I. pseudacorus*,* I. walleriana*,* K. uvaria*,* L. clethroides*,* S. hybrida*,* L. martagon*,* D. seguieri*). For species producing less nectar (*A. archangelica*,* P. caeruleum*,* P. russeliana*), nectar was pooled from up to five flowers, ensuring all were located on the same shoot. Collected nectar was transferred into sterile Eppendorf tubes and stored at −20 °C.

### Flower visitors observations

To assess insect visitors and their visitation frequency, the method described by Zych et al.^50^ was adapted. Selected flowers were recorded for ten 15-min sessions, using digital cameras mounted on tripods positioned approximately 1 m away so as not to influence insects visits. Each plant species was recorded for at least 150 min per day for five days. Recordings were conducted between 10:00 and 16:00 CEST which corresponds to roughly 2.5–3.5 h before and after solar noon (around 09:20–15:20 solar time), coinciding with peak animal activity, and only under suitable weather conditions (avoiding rainy and windy days). In the laboratory, the footage was reviewed and each insect that visited a flower and contacted nectar was counted. Insects were classified into the following groups: honeybee, bumblebee, hoverflies, ants, flies, solitary bees, other Hymenoptera, and other. Visitation frequency was calculated per hour.

### Nectar chemistry analysis

In the laboratory, nectar was diluted with water to a volume of 50 µl (10 µl of nectar + 40 µl of water). The sample was filtered through spin columns using a 0.4 μm pore size membrane filter before injection. The filtrate was then loaded into the insert. An Agilent 1260 Infinity Series HPLC system with an autoinjector, refrigerated autosampler compartment, thermostatted column compartment, quaternary pump with an inline vacuum degasser, and refractive index detector was used. A ZORBAX Carbohydrate Analysis Column (4.6 mm× 250 mm, 5 μm) was used for sugar separation and analysis. A 10 µl aliquot sample or standard solution was injected. The separation was conducted at 30◦C with the mobile phase comprising acetonitrile: water (70:30, v/v) at a flow rate of 1.4 ml/min. The analytical data were integrated using the Agilent OpenLab CDS ChemStation software for liquid chromatography (LC) systems. Identification of sugars was performed by comparing the retention times of individual sugars in the reference vs. test solution. The content of glucose, fructose, and sucrose was assayed based on comparisons of peak areas obtained for the samples.

Collected nectar was also analyzed for the composition of the nectar’s amino acids with the use of HPLC. After thawing the samples to an ambient temperature, the nectar was diluted to a volume of 20 µL (10 µL of nectar was mixed with 10 µL of distilled water). The sample was filtered through a spin column with a 0.4 μm pore size membrane filter (A&A Biotechnology, Poland) before injection by centrifugation for 2 min at 9000 g (relative centrifugal force). The filtrate was loaded into the insert and analyzed by an HPLC. The samples were analyzed using an Agilent Technologies 1260 Infinity series system consisting of a 1260 Infinity Agilent Quaternary pump G1311B, a 1260 Infinity Diode Array Detector (DAD) G1315D, a 1260 Infinity Fluorescence Detector (FLD) G1321B, a 1260 Infinity ALS G1329B Automated Sample Injector, a 1290 Infinity Autosampler Thermostat G1330B and a thermostatted column oven 1290 Infinity TCC G1316C. The system was controlled by Agilent OpenLab ChemStation software. The analysis of amino acids in 10 µL aliquots of nectar collected from flowers was performed by gradient HPLC using an Agilent Zorbax Eclipse Plus C18 (4.6 × 150 mm, 5 μm) column with a guard, i.e. Agilent Zorbax Eclipse Plus C18 (4.6 × 12.5 mm, 5 μm). The extracts, containing primary and secondary amino acids were pre-column derivatized with o-phtalaldehyde (OPA) and 9-fluorenylmethyl chloroformate (FMOC) reagent. An injector program was used for the derivatization. Following derivatization, a mixture of each sample was injected into a pre-equilibrated column operated at 40 °C. The primary (OPA-derivatized) amino acids were monitored at 388 nm by DAD while the secondary (FMOC-derivatized) amino acids were monitored by FLD, at an excitation wavelength of 266 nm and an emission wavelength of 305 nm. Mobile phase A was 40 mM NaH2PO4 (pH 7.8 adjusted using 10 M NaOH solution), while mobile phase B was acetonitrile: methanol: water (45:45:10. v/v/v). The following gradient profile was seen: 0–5 min: 0% B t- 10% B; 5–25 min: 10% B − 40.5% B; 25–30 min: 40.5% B − 63% B; 30–35 min: 63% B − 82% B; 35–37 min: 82% B − 100 B; 37–39 min: 100% B; 39–40 min: 100% B- 0% B; 40 43 min: 0% B. A flow rate of 1 mL/min was used.

### Mycobiome analysis

Total environmental DNA was isolated using Fast DNA Spin Kit for Feces (MP Biomedicals) using manufacturer instructions with changes in the following steps:

In step 1. “In a 2 mL Lysing Matrix E tube, add 500 mg feces sample, 825 µL Sodium Phosphate Buffer, and 275 µL of PLS solution. Shake to mix. Vortex 10–15 seconds” we did not use Lysing Matrix Eppendorf tubes.

In step 4. “Homogenize samples in the FastPrep 24 instrument at setting 6.0m/s for 40 seconds” FastPrep 24 instrument homogenization was changed to microwave interval homogenization (800 W, 5 s of heating, 10 s rest, repeated 3 times).

In steps 8 and 9: “8. While samples are centrifuging, add 1 mL of Binding Matrix Solution to a clean 15 mL conical tube (not supplied). 9. Transfer supernatant to the Binding Matrix Solution in the 15 mL conical. Shake gently by hand to mix, then place on a shaker/rocker for 3–5 minutes.” A 15 mL tube was changed to 2 mL Eppendorf tube and samples were vortexed for 4 min instead of being shaken.

Other steps have not been changed. DNA isolation was conducted with five technical repetitions for each variant (isolated and open flowers) of each plant species. Since some of the repetitions did not contain enough DNA for further analyses, we pulled together DNA isolated from nectar derived from one flower and one variant into one probe.

We prepared PCR mixtures using total isolated DNA and following primers (with Illumina adaptors): for 16 S region we used V3 (TCGTCGGCAGCGTCAGATGTGTATAAGAGACAGCCTACGGGnGGCwGCAG) and V4 (GTCTCGTGGGCTCGGAGATGTGTATAAGAGACAGGACTAChvGGGTATCTAATCC) and for ITS2 region we used ITS3 (TCGTCGGCAGCGTCAGATGTGTATAAGAGACAGGCATCGATGAAGCGCAGC) and ITS4 (GTCTCGTGGGCTCGGAGATGTGTATAAGAGACAGTCCTCCGCTTATTGATATGC) oligonucleotides. Each reaction mixture contained: 20 µL of KAPA HiFi polymerase, 6 µL of both primers from each pair and 14 µL of isolated DNA. ITS PCR protocol was identical as in Okrasińska et al.^51^. All reactions were prepared in four technical repetitions in order to minimize bias, and then pulled together. Two negative controls containing sterile water were prepared and all of the actions described above were performed with them. The amplicons were prepared and sent on the base of nectar and water to Genomed (Warsaw, Poland) for library preparation and Illumina MiSeq paired end sequencing and bioinformatic analysis of obtained results.

Although nectar volume per flower was generally sufficient, DNA yield from individual nectar samples was often low and in several cases insufficient for reliable amplification and sequencing. To obtain enough template DNA, nectar from multiple flowers within the same site and treatment was pooled prior to DNA extraction. This procedure resulted in a reduced number of independent nectar samples for mycobiome analyses. DNA extraction and amplicon sequencing were accompanied by extraction and PCR negative controls. After sequence processing, we removed amplicon sequence variants (ASVs) occurring in negative controls and excluded any samples whose community profiles were strongly affected by these contaminant ASVs. The final dataset therefore comprised 12 high-quality samples, which were used in all mycobiome diversity analyses.

### Statistical analysis of survey data

All analyses were performed in R^52^. To assess the alpha diversity of nectar-associated microbial communities, two complementary indices were calculated: Shannon diversity and the Chao1 richness estimator. Shannon diversity was used as the primary metric for statistical analysis and visualization, as it reflects both species richness and evenness, providing a comprehensive measure of community complexity. Chao1 was included to estimate total richness, including rare or undetected taxa, which is particularly relevant in systems with uneven sequencing depth or low detection rates. Both indices were calculated from taxon abundance tables derived from ITS amplicon sequencing using the vegan^53^ and phyloseq packages^54^ in R^52^.

Due to the limited sample size (final *n* = 12) generalized linear mixed models were not used to analyze the relationships between studied factors and microbial diversity. Spearman correlation was used to examine associations between microbial diversity and nectar chemistry, as well as between diversity and insect visitation. Kruskal–Wallis tests assessed the effects of flower traits (size, position, and accessibility) on diversity and the abundance of individual fungal families. Additionally, correlations between pollinator groups and fungal abundance were tested. For each fungal family and floral trait, we first performed Kruskal–Wallis tests. Given the large number of families, we did not apply a global correction across all Kruskal–Wallis tests in order to avoid excessive inflation of Type II error and the risk of overlooking biologically meaningful patterns. When Kruskal–Wallis test was significant (*p* < 0.05), we conducted Dunn’s post hoc tests to identify which levels of the trait differed, using Bonferroni adjustment within each set of pairwise comparisons. In all of the analyses taxa present both in nectar and in the negative control as contamination were excluded.

To determine whether nectar amino acid and sugar composition differed between open and restricted flowers, we first calculated average concentrations for each species and access type. Only species with at least two replicates per access type were included in the statistical analysis to ensure reliability. Differences between open and restricted flowers were assessed using Welch’s *t*-test, which was selected due to unequal sample sizes and variances.

## Results

### Nectar properties

**Fig. 1:**
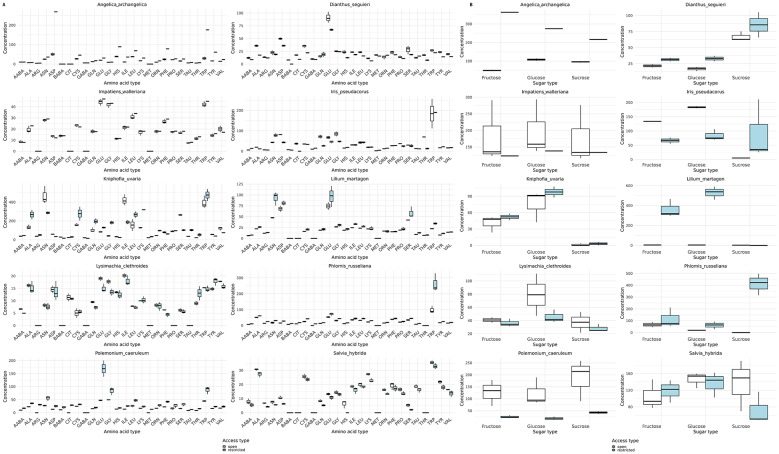
Boxplots displaying the concentration of amino acids [**A**] and sugars [**B**] in nectar across flowers open (white) for flower visitors and with restricted access (blue). [**A**] Each panel represents a different plant species, with amino acid concentrations shown on the y-axis (pmol/μl) and amino acid types on the x-axis. The abbreviations for amino acids are as follows: AABA (alphaaminobutyric acid), ALA (alanine), ARG (arginine), ASN (asparagine), ASP (aspartic acid), BABA (beta-aminobutyric acid), CIT (citrulline), CYS (cysteine), GABA (gamma-aminobutyric acid), GLN (glutamine), GLU (glutamic acid), GLY (glycine), HIS (histidine), ILE (isoleucine), LEU (leucine), LYS (lysine), MET (methionine), ORN (ornithine), PHE (phenylalanine), PRO (proline), SER (serine), TAU (taurine), THR (threonine), TRP (tryptophan), TYR (tyrosine), VAL (valine). [**B**] Boxplots displaying the concentration of sugars recorded in nectar. Each panel represents a different plant species, with sugar concentrations shown on the y-axis (pmol/μl) and sugar type on the x-axis. The central line of each box represents the median value, while the lower and upper edges of the box correspond to the first and third quartiles (interquartile range, IQR). Whiskers extend to the smallest and largest observations within 1.5 times the IQR from the quartiles, while individual points outside this range are considered outliers.

Nectar samples from 48 flowers, from ten plant species were collected. Each sample was examined for amino acid and sugar content, revealing 21 amino acids and three sugar types that varied in both concentration and proportion. In open flowers, tryptophan was the most abundant amino acid (491 ± 109 pmol/µl, 22.8% ± 3.6), while α-aminobutyric acid was the least abundant (12.2 ± 2.6 pmol/µl, 0.9% ± 0.1). Similarly, in isolated flowers, tryptophan predominated (422 ± 71.8 pmol/µl, 19.8% ± 3.8), and β-aminobutyric acid was lowest (12.3 ± 1.2 pmol/µl, 1.1% ± 0.4); Table 2 A (Supplementary materials) and Fig. 1A. Across species, nectar sugar concentrations were generally higher in restricted flowers. In contrast, amino acid composition showed no consistent pattern, with some species (e.g., *D. seguieri*, *I. pseudacorus*) exhibiting higher amino acid levels in open flowers, while others (e.g., *A. archangelica*, *L. martagon*) had elevated levels in restricted flowers Table 2B (Supplementary materials). Overall, the highest total amino acid content was observed in the nectar of open *P. caeruleum* flowers (3160 pmol/µl), and the lowest in isolated *D. seguieri* flowers (1727 pmol/µl). Every sample contained sucrose, glucose, and fructose, with sucrose dominating in all cases; its highest concentration was found in isolated *A. archangelica* flowers (766 pmol/µl) and the lowest in open *I. walleriana* flowers (263 pmol/µl). Glucose and fructose levels were similar across samples, though both were noticeably lower in isolated flowers. Detailed nectar data (amino acids and sugar content) are summarized in Table 2B (Supplementary materials) and Fig. 1B.

### Pollinators analysis

During 843 min of recording we noted 1502 insects’ visits. The most frequently visited plant species was *K. uvaria* with 442 visits, while the least *D. seguieri* with 11 visits. The most frequently recorded visitors were bees overall (80.5% of visits, combining all bee groups, then other Hymenoptera (9.4%) and honeybees (8.3%). Detailed visitors analysis is shown in Table 3 (Supplementary materials).

### Microbiome analysis

After sequencing using the Illumina Miseq platform, we obtained 479 fungal OTUs present in nectar collected from the studied plants. Analyses linking nectar properties to mycobiome diversity were performed on the subset of samples (*N* = 12) that combined sufficient DNA yield, passed all quality-control filters, and had both nectar chemical property measurements and high-quality sequencing data available. Identified fungi belong to the following families: Aspergillaceae, Bionectriaceae, Bondarzewiaceae, Buckleyzymaceae, Bulleribasidiaceae, Cladosporiaceae, Coniochaetaceae, Coniosporiaceae, Coniothyriaceae, Cordycipitaceae, Cucurbitariaceae, Cyphellaceae, Debaryomycetaceae, Dermateaceae, Diademaceae, Dipodascaceae, Dissoconiaceae, Dothidotthiaceae, Erysiphaceae, Filobasidiaceae, Herpotrichiellaceae, Hydnaceae, Hypoxylaceae, Lachnaceae, Leptosphaeriaceae, Malasseziaceae, Melanommataceae, Metschnikowiaceae, Microascaceae, Mollisiaceae, Mortierellaceae, Mrakiaceae, Mycosphaerellaceae, Nectriaceae, Orbiliaceae, Paradevriesiaceae, Phacidiaceae, Phanerochaetaceae, Pleosporaceae, Pleurotaceae, Polyporaceae, Pseudeurotiaceae, Pseudodidymellaceae, Rhynchogastremaceae, Rhytismataceae, Saccharomycodaceae, Saccotheciaceae, Sclerotiniaceae, Sistotremataceae, Sordariaceae, Sporidiobolaceae, Sporocadaceae, Sporormiaceae, Strophariaceae, Symmetrosporaceae, Trichomeriaceae, Trichomonascaceae, Trichosporonaceae, Umbelopsidaceae, Wickerhamomycetaceae. The majority of hits within fungi were annotated to the Phylum Ascomycota (402 OTUs), followed by Basidomycota (33 OTUs), Mortierellomycota (15 OTUs), Mucoromycota (26 OTUs) and unassigned (3 OTUs).

### Flower traits and mycobiome diversity

**Fig. 2:**
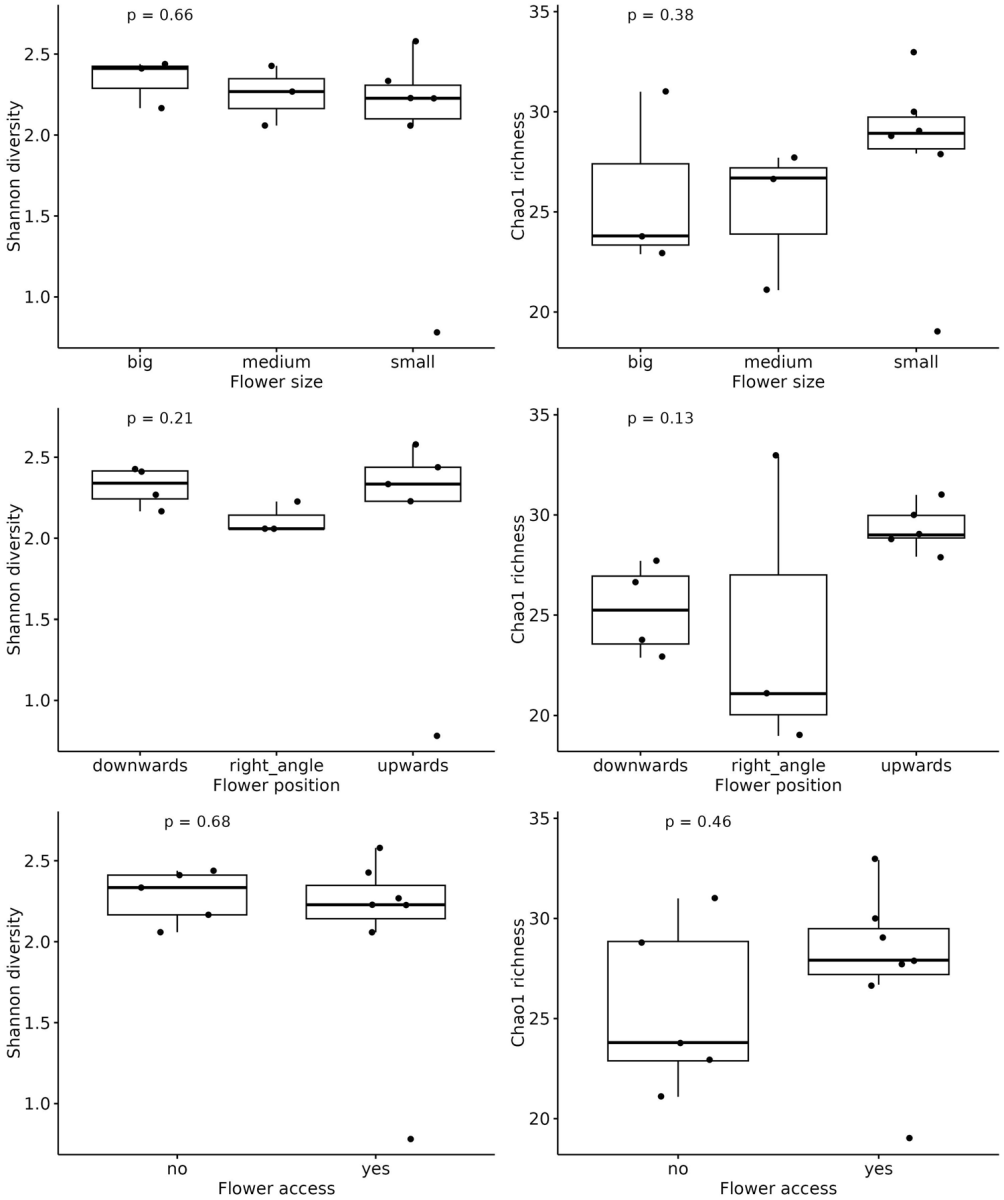
Boxplots illustrating the variation in nectar microbial diversity (Shannon diversity and Chao1 richness) in relation to studied traits. Panels show microbial diversity in response to flower size, position, and accessibility (open for flower visitors and with restricted access) and significance level. No significant differences were observed based on these categorical traits (Kruskal- Wallis test, all p > 0.2). All figures include jittered data points overlaid on boxplots.

Shannon mycobiome diversity did not differ among flower size categories or flower positions. Similarly, Chao1 richness showed no variation with flower size or flower position (Fig. 2). Although overall mycobiome diversity did not vary with floral traits, the occurrence of Mollisiaceae was associated with flower size (Table 4 Supplementary Materials).

### Nectar properties and mycobiome diversity

Overall, there were no significant correlations between any amino acid category (including alkaline amino acids: arginine, histidine, lysine, ornithine, and citrulline; acidic amino acids: aspartic acid and glutamic acid; neutral amino acids: asparagine, glutamine, serine, threonine, cysteine, and tyrosine; aromatic amino acids: phenylalanine, tyrosine, tryptophan, and histidine; aliphatic amino acids: alanine, glycine, leucine, isoleucine, valine, proline, and methionine; proteinogenic amino acids: aspartic acid, glutamic acid, alanine, cysteine, glycine, serine, threonine, tyrosine, arginine, asparagine, glutamine, histidine, lysine, proline, isoleucine, leucine, methionine, phenylalanine, tryptophan, and valine; and non-proteinogenic amino acids: ornithine, citrulline, taurine, α-aminobutyric acid, β-aminobutyric acid, and γ-aminobutyric acid) and mycobiome diversity. No relationships were observed between amino acid concentrations and fungal diversity. Correlations between Shannon diversity or Chao1 richness and the different amino acid categories were weak (Shannon: ρ from − 0.36 to 0.30; Chao1: ρ from − 0.12 to 0.27) and non-significant (all *p* > 0.24; Fig. 2 Table 4 Supplementary Materials).

**Fig. 3:**
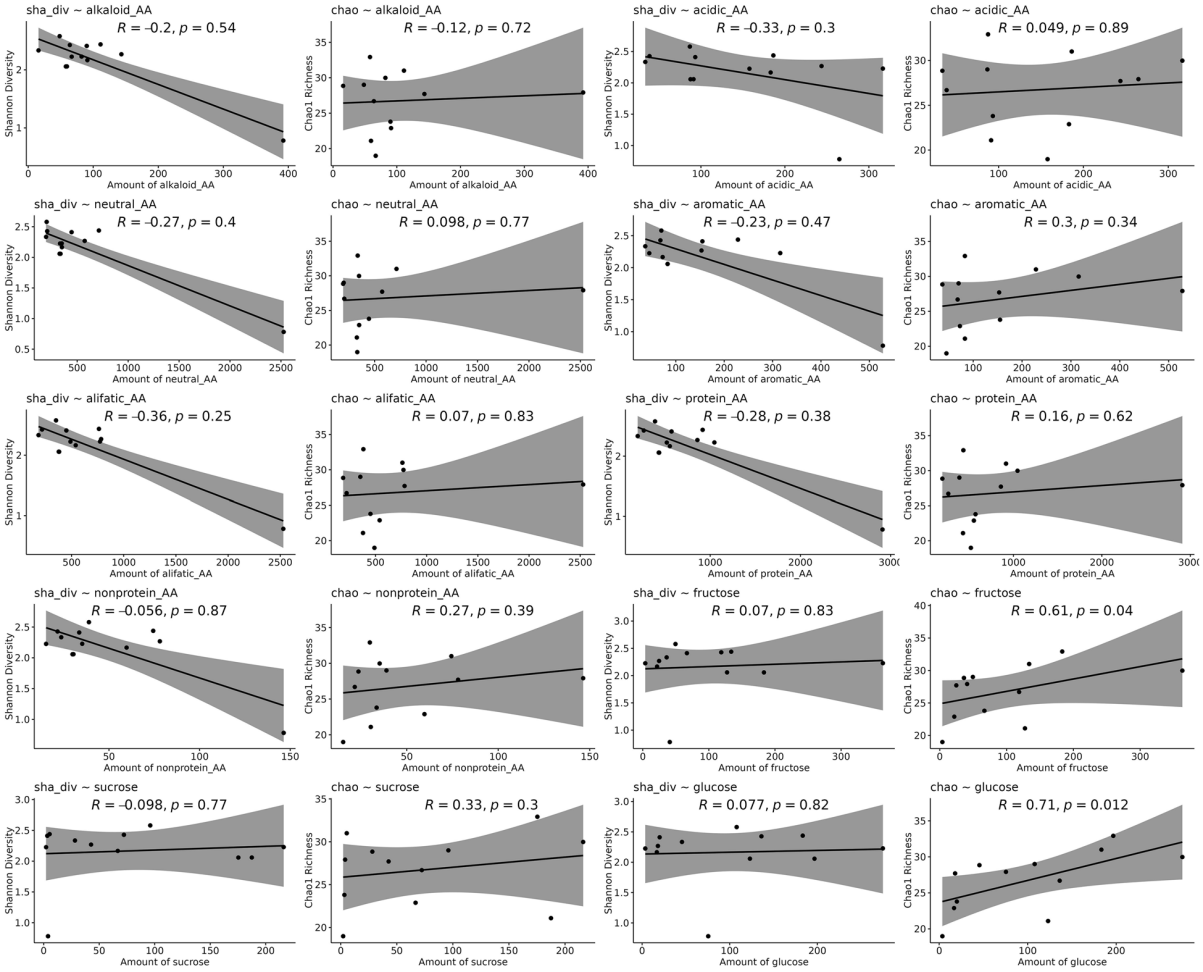
Relationships between nectar microbial diversity (Shannon diversity and Chao1 richness) and nectar chemistry (amino acids and sugars amount). Each panel presents a scatterplot with a fitted trend line, showing how microbial diversity varies with the presence or absence of nectar compounds, including amino acid classes (alkaline amino acids: arginine, histidine, lysine, ornithine, and citrulline; acidic amino acids: aspartic acid and glutamic acid; neutral amino acids: asparagine, glutamine, serine, threonine, cysteine, and tyrosine; aromatic amino acids: phenylalanine, tyrosine, tryptophan, and histidine; aliphatic amino acids: alanine, glycine, leucine, isoleucine, valine, proline, and methionine; proteinogenic amino acids: aspartic acid, glutamic acid, alanine, cysteine, glycine, serine, threonine, tyrosine, arginine, asparagine, glutamine, histidine, lysine, proline, isoleucine, leucine, methionine, phenylalanine, tryptophan, and valine; and non-proteinogenic amino acids: ornithine, citrulline, taurine, α-aminobutyric acid, β-aminobutyric acid, and γ-aminobutyric acid) and sugars (fructose, sucrose, glucose). The x-axes indicate the amount of specific amino acids compounds. The y-axes represent either Shannon diversity or Chao1 richness of the nectar microbial community in each sample. Correlation coefficients (Spearman’s ρ) and associated p-values are provided for each relationship.

Sugar composition showed associations with mycobiome diversity. Chao1 richness increased with both fructose and glucose concentrations, whereas sucrose concentration was not related to either index (Fig. 3; Table 4 Supplementary Materials). Shannon diversity was not correlated with any sugar traits.

Several correlations were observed between fungal families’ abundance and nectar chemistry. Phanerochaetaceae abundance increased with higher concentrations of alkaline, aliphatic, neutral and non-protein amino acids, and additional positive correlations with neutral amino acids were observed for Coniochaetaceae and Mollisiaceae. Moreover, a number of families, particularly Cladosporiaceae, Leptosphaeriaceae, Pleosporaceae and Malasseziaceae, were positively associated with fructose and/or glucose concentrations, whereas Rhytismataceae was negatively associated with glucose (Table 4 Supplementary Materials).

### Influence of flower visitors on microbiome diversity

**Fig. 4:**
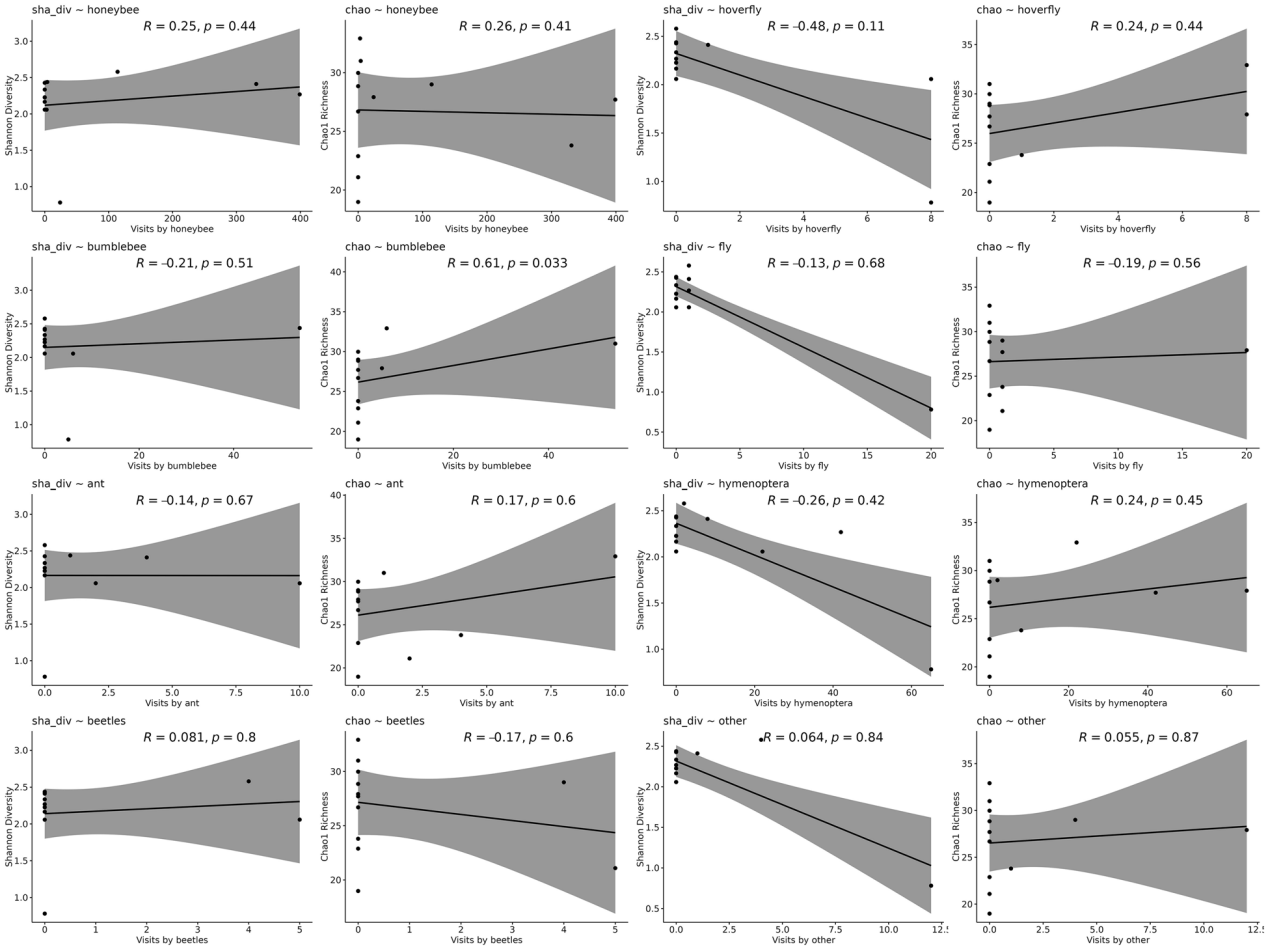
Relationships between nectar microbial diversity and the number of visits by different flower visitors groups. Each panel presents a scatterplot with a fitted trend line, showing how microbial diversity (measured by Shannon diversity index (sha_div) and Chao1 richness (chao)) varies with flower visitor visitation rates. The x-axes indicate the number of visits by specific groups: honeybees, hoverflies, bumblebees, flies, ants, hymenopterans, beetles and other. The y-axes represent either Shannon diversity or Chao1 richness of the nectar microbial community in each sample. Correlation coefficients (Spearman’s ρ) and associated p-values are provided for each relationship.

Neither Shannon diversity nor Chao1 richness varied with flower accessibility (Fig. 1). Overall, pollinator visitation rates were generally weakly related to fungi diversity:, only bumblebee visitation was positively correlated with Chao1 richness, while no relationships were found for Shannon diversity (Fig. 4; Table 4 Supplementary Materials). Nonetheless, the abundance of several fungal families covaried with visitation by specific pollinator groups. In particular, multiple families were positively associated with fly visits, and further positive correlations were detected between certain families and visits by hoverflies, ants and other hymenopteran visitors (Table 4 Supplementary Materials).The occurrence of Coniochae and Mollisiaceae was associated with flower accessibility (Table 4 Supplementary Materials). indicating that this family may respond to floral openness or restrictions in nectar exposure.

## Discussion

Our study indicates that fungal diversity in floral nectar may be influenced by the concentration of specific sugars, particularly fructose and glucose, highlighting their potential role in shaping the nectar mycobiome. While other nectar properties, floral traits, and flower visitors had little effect on overall diversity, the relative abundance of certain fungal families was associated with specific features, such as floral morphology, nectar chemistry, and visitor presence. We also recorded differences in sugar and amino acids presence in flowers with open and restricted visitors access. To some extent, these differences are likely linked to the presence and activity of mycobiome introduced primarily through animal visits.

Nectar serves as a habitat for diverse microbial communities, with plant species-specific properties regulating fungal colonization^55,56^. In our study, the family Mollisiaceae varied with flower size, suggesting this floral trait may influence its abundance. This effect could be related to increased time pollinators spend accessing nectar or the extent of contact between the pollinator’s body and the nectar or its surface, factors known to shape microbiome diversity^20^. Additionally, flower size may influence the likelihood of pollinators defecating on floral surfaces, which can in turn affect fungal diversity^28^.

In our experiment, floral traits did not appear to influence fungal diversity, and most fungal families detected showed no clear response to these features. Similar findings were reported by Adler et al.^21^, who observed that floral size and shape, number of open flowers, nectar production, and inflorescence height did not explain interspecific variation in floral microbiomes. In their study, the only trait associated with microbiome composition was the total number of reproductive structures per inflorescence. It is also worth noting that several fungal families found in the nectar of the studied species are not considered typical nectar specialists. Their presence is likely due to environmental contamination, surface colonization, or incidental transfer by pollinators, rather than adaptation to the nectar environment.

In contrast to floral traits, our results highlight the role of nectar chemistry, particularly glucose concentration, in shaping fungal diversity. Similarly, in African *Watsonia*, yeast density was found to decrease with increasing sugar content and nectar concentration^36^. The influence of specific sugar concentration on mycobiome may reflect the level of tolerance to high-sugar conditions and competition among sugar-adapted fungi^2,8^. The presence of the fungal family Pleosporaceae was positively correlated with both glucose and fructose concentrations, whereas Sclerotiniaceae and Didymellaceae showed negative correlations with glucose. The strong correlations between fructose and abundance of families such as Cladosporiaceae, Leptosphaeriaceae, and Malasseziaceae may reflect the ability of these taxa to thrive in high-sugar environments, which often require efficient osmoregulation and specialized metabolism^57^. Glucose, which strongly correlated with Pleosporaceae and several other families, is often a dominant energy source in nectar, potentially favoring generalist fungi capable of rapid sugar assimilation^14^. The recorded influence of glucose and fructose may be related to the fact that both of these two sugars make water activity lower and due to that fact they may make the nectar environment harder to inhabit^58^. The positive correlation of Phanerochaetaceae presence with multiple amino acid groups (alkaloid, aliphatic, nonprotein, and neutral amino acids) indicates that they might provide nutrients supporting the growth of certain filamentous fungi, as previously suggested for nectar yeasts^2,8^.

A wide range of fungal families showed no correlation with nectar sugar or amino acid composition and concentration. Although nectar traits are recognized as important drivers of microbial diversity, their effects can be difficult to disentangle from other plant-related factors. Even strains of the same microbial species can exhibit substantial variation in growth rates when exposed to identical nectar sources^20,32^. Moreover, microbial community assembly in nectar may be strongly shaped by priority effects, whereby early-arriving microbes, such as certain bacteria or yeasts, can inhibit the establishment or growth of subsequent colonizers^59,60^.

As in the case of recorded relations between fungal families and flower traits, also in this case — none of the fungal families that showed a response to nectar properties appear to be nectar specialists. Pleosporaceae representatives, however, are frequently detected in floral microbiome studies, since they are the part of the plants’ mycobiome^61^. Herrera et al. (2010, 2013) reported filamentous fungi from the order *Pleosporales* on flower surfaces and potentially within nectar. In contrast, Sclerotiniaceae and Didymellaceae are not considered typical members of nectar mycobiome. Members of Sclerotiniaceae, such as *Botrytis* and *Sclerotinia*, are well-known necrotrophic pathogens that infect a broad range of floral and vegetative tissues^22^. Similarly, members of Didymellaceae are common plant-associated fungi found on leaves and stems or as endophytes^62^. Although some members have occasionally been reported in floral microbiome studies^63^, there is limited evidence to suggest that this family is adapted to the nectar environment or plays an active ecological role there. Based on our results and the taxonomic richness of Sclerotinaceae and Didymellaceae in our samples we think that their presence in nectar is not accidental. This may be one of the ways of the dispersal of these fungi. When their diaspores or hyphae are present in the nectar of plants they inhabit, there is a high possibility of transporting these fungi to other plants. Nevertheless, methods used by us do not allow for checking whether spores or mycelium of these fungi is viable in nectar conditions and test this hypothesis. However, it is also important to stress that limited attention has been paid so far to the fungal endophytes of flowers^64,65^, while some may improve the host plants’ growth^66^. Most published data focus on the presence of fungal pathogens or mycotoxin producers in the flowers of economically-important plants^67^ and literature there in].

Flowers receive a variety of animal visitors including both pollinators and non-pollinating florivores that regularly share their own surface and internal microbiota with the nectar^8,20,36,38,68–71^. Our results also showed that the family Mollisiaceae and Coniochaetaceae differed significantly across flowers open for flower visitors and with restricted access, potentially reflecting dispersal differences based on pollinator entry routes. Other studies revealed also that ascomycetous yeasts were autochthonous members of the communities while species in the *Metschnikowia* clade, the *Starmarella* clade, and the genera *Debaryomyces* and *Zygosaccharomyces* were associated with flower visitors presence^15^. However, in our study the frequency of specific visitors was not correlated to the fungal diversity in the nectar. Only some families, like Pseudeurotiaceae and Strophariaceae showed positive associations with fly visitation, indicating that these fungi may be dispersed or promoted by fly activity. Members of these families often produce airborne spores or occur in saprotrophic habitats, making them likely candidates for mechanical transfer by flies^72^. They frequently move between decaying material and flowers, and can act as effective fungal dispersers, especially for non-specialist or opportunistic taxa^14,70^. However, their detection in nectar may not indicate active colonization; instead, it may reflect incidental introduction through contact with floral surfaces or nearby substrates. On the other hand, we hypothesize that transport of these fungi diaspores to nectar can act like a short stop of their journey between different substrates they can inhabit and successfully colonize since fungal spores can act like a resting stage of the life cycle^73^. Moreover, the generally weak influence of visitors on overall fungal diversity may be partially explained by a dilution effect, occurring in systems with high floral resource availability, where microbial inoculation is spread across many potential hosts.

It is important to highlight that our study reports the presence of several hyphomycete genera not previously documented in floral nectar. These fungi may possess unique adaptations to the nectar environment, and their biology warrants further investigation. For example, we detected *Beauveria bassiana*, a species known for its entomopathogenic and endophytic properties^74,75^. Its presence in floral nectar may represent a previously overlooked part of its life cycle and deserves broader investigation. A deeper understanding of the factors shaping fungal diversity at this small scale could shed new light on the role of microbial third-party players in pollination mutualisms.

Flowers isolated from visitors provide an essential baseline: if nectar fungal communities in isolated flowers change less, or differently, than in visited flowers, this supports pollinator access as a key driver of nectar mycobiome dynamics under field conditions. We found clear differences in nectar chemistry between open and restricted flowers that are consistent with visitor-mediated microbial introduction. Sugar concentrations were higher in restricted flowers, in line with reduced microbial colonization, whereas open flowers likely experienced greater sugar depletion due to yeast and fungal metabolism^8,36,76^. Amino acid patterns were more variable, but also compatible with microbial effects: some amino acids (e.g., threonine, alanine) were higher in open flowers, while others were enriched in restricted flowers, reflecting that nectar yeasts can both consume and produce amino acids in a species-specific manner^19,71,77^.

Our study has several limitations. Microbial presence in nectar was generally low, likely due to the botanical garden setting during peak bloom, where high plant diversity may have diluted pollinator visits and limited microbial transfer. Individual plants in botanic garden collection are rather grouped by species and not interspersed by other taxa, which may have promoted similarity in nectar mycobiomes, especially given the horizontal transmission of most endophytes^78^. Methodological factors may have also contributed: single-time-point sampling, small nectar volumes, and limitations of DNA extraction and primer specificity may have reduced detection of low-abundance taxa. Moreover, part of focal plant species used in the study are non-native ornamentals, growing outside their native ranges. Such mixed, native and non-native species communities are increasingly common as cities’ areas grow. It is therefore important to understand the nectar properties and floral microbiomes of these communities, as they can make a substantial contribution to urban foraging resources. At the same time, pollinator assemblages and abiotic conditions in the plants’ native ranges may differ from those in our urban system, and we currently lack direct comparisons of nectar microbiomes between native and introduced ranges of the same species. We thus interpret our results specifically in the context of urban plant–pollinator–microbe interactions.

We are also aware of the fact that our study may have detected some fungal OTUs that are not metabolically active in the nectar. Nevertheless, most of the fungi are not culturable on standard, artificial media^27^. With low volumes of nectar samples we were not able to divide it into smaller portions and conduct a proper culture based study on different media suitable for growth of different fungi. Additionally, the unmeasured bacterial communities may contribute to the patterns we observed in fungal diversity and composition. Although our study focuses only on fungi, integrating both fungal and bacterial components of the floral microbiome is an important direction for future research. Despite these constraints, our study highlights the presence of filamentous fungi in nectar, a group previously overshadowed by yeasts, and offers a starting point for future research on fungal and plant interactions.

## Supplementary Information

Below is the link to the electronic supplementary material.

Supplementary Material 1

Supplementary Material 2

Supplementary Material 3

Supplementary Material 4

## Data Availability

Data will be available at [https://doi.org/10.58132/TXPN99](https:/doi.org/10.58132/TXPN99)We deposited all of the raw sequences generated during our study in Genbank Sequence Read Archive under Bioproject PRJNA1332572.

## Declarations

### Generative AI and AI-assisted technologies in the writing process

During the preparation of this work the authors used chatgpt/OpenAI in order to edit the writing. After using this tool, the authors reviewed and edited the content as needed and take full responsibility for the content of the publication.
